# Poly-l-Lactic Acid (PLLA)-Based Biomaterials for Regenerative Medicine: A Review on Processing and Applications

**DOI:** 10.3390/polym14061153

**Published:** 2022-03-14

**Authors:** Elisa Capuana, Francesco Lopresti, Manuela Ceraulo, Vincenzo La Carrubba

**Affiliations:** 1 Department of Engineering, University of Palermo, RU INSTM, Viale delle Scienze, 90128 Palermo, Italy; elisa.capuana@unipa.it (E.C.); manuela.ceraulo@unipa.it (M.C.); vincenzo.lacarrubba@unipa.it (V.L.C.); 2 ATeN Center, University of Palermo, Viale delle Scienze, 90128 Palermo, Italy

**Keywords:** poly-l-lactic acid (PLLA), tissue engineering, regenerative medicine

## Abstract

Synthetic biopolymers are effective cues to replace damaged tissue in the tissue engineering (TE) field, both for in vitro and in vivo application. Among them, poly-l-lactic acid (PLLA) has been highlighted as a biomaterial with tunable mechanical properties and biodegradability that allows for the fabrication of porous scaffolds with different micro/nanostructures via various approaches. In this review, we discuss the structure of PLLA, its main properties, and the most recent advances in overcoming its hydrophobic, synthetic nature, which limits biological signaling and protein absorption. With this aim, PLLA-based scaffolds can be exposed to surface modification or combined with other biomaterials, such as natural or synthetic polymers and bioceramics. Further, various fabrication technologies, such as phase separation, electrospinning, and 3D printing, of PLLA-based scaffolds are scrutinized along with the in vitro and in vivo applications employed in various tissue repair strategies. Overall, this review focuses on the properties and applications of PLLA in the TE field, finally affording an insight into future directions and challenges to address an effective improvement of scaffold properties.

## 1. Introduction

Tissue engineering (TE) is a multidisciplinary field that encompasses life sciences and engineering to develop biological substitutes that replace, repair, and improve the functions of tissues [1,2,3]. Scaffolds, along with cells and growth factors, play a crucial role in achieving the purpose of TE. An ideal scaffold should mimic the native extracellular matrix (ECM), an endogenous substance that surrounds cells and provides spatial and mechanical signals aiding cellular development and morphogenesis [4]. Scaffolds need to be biodegradable materials whose degradation must be synchronic with the tissue growth [5,6]. Therefore, the actual challenge of TE is to fabricate scaffolds with adequate physical and biological properties leading to proper cell growth while ensuring appropriate mechanical properties for the in vivo environment [7,8]. Among the biodegradable polymers used for tissue engineering, poly-l-lactic acid (PLLA) has been widely studied because of its interesting mechanical properties and tailorable biodegradability [9]. As a result, it can maintain mechanical and structural integrity during in vitro and in vivo applications while supporting tissue formation [10,11,12]. PLLA belongs to the PLA family, and, compared to PDLA (created through the polymerization of D-lactide), it exhibits higher crystallinity, chemical stability, and degradation resistance to enzymes and, as a consequence, a much longer resorption time [1,13,14,15]. Moreover, the degradation of PLLA produces L-lactic acid, which is harmless to the human body, while D-lactic acid, produced by PDLA, is slightly harmful [16]. In addition, PLLA is synthesized from eco-sustainable processes, which do not use oil sources or poorly cleaned catalysts, and is approved by the FDA for its non-cytotoxicity, suggesting that PLLA-based scaffolds could effectively promote tissue regeneration. However, PLLA is a polyester that can be degraded by hydrolysis to form acidic byproducts that alter the local pH and thus impair the differentiation of cells seeded into the scaffolds [17]. Biochemical or physical processes can induce superficial modifications, thus influencing the hydrophobicity of the PLLA and improving cell adhesion and protein adsorption [18,19]. A similar response is achieved by introducing growth factors into the material, thus facilitating tissue repair both in vitro and in vivo [20,21]. Tissue engineering has undergone remarkable progress, developing scaffolds with ideal properties using composite or hybrid systems. PLLA is often combined with other biomaterials, such as natural and synthetic polymers and inorganic materials, surpassing the limit of using a single polymer. For functional structures, the aim is to provide sufficient architecture and rigidity for the tissue to replace. There are many reports of manufacturing PLLA scaffolds using electrospinning [22], additive manufacturing [23], particulate-leaching [24], and phase separation [25] for various tissue engineering applications.

Another notable application of scaffolds is their use as supporting materials for different drug loadings, allowing for the sustained and controlled release of drugs over the desired time period [26,27]. Several engineered systems have been investigated for this purpose, including natural materials such as gelatin [28], zein [29], bovine serum albumin (BSA) [30], kefiran [31,32] and chitosan [33] or synthetic polymers such as polycaprolactone (PCL) [34], poly (lactic-co-glycolic acid) (PLGA) [35], and PLA [36]. For these therapeutic applications, a low rate of biodegradation is required [30,37], as observed for PLLA. Hence, it is a suitable candidate for a prolonged drug delivery system [10,38,39].

Several interesting reviews in the scientific literature highlight the use of PLA [40,41,42] as a biomaterial for scaffold fabrication, but there is a lack of reviews on the same topic for PLLA. Since PLLA is an increasingly used material in tissue engineering, a review of its essential properties would help to choose such material for a variety of applications. This review summarizes and discusses the main properties of PLLA-based scaffolds and the modifications proposed to improve the performance of these systems. A literature analysis about hybrid scaffolds fabrication and properties combining PLLA and other biomaterials is also presented. In addition, PLLA-based scaffolds used for bone, cartilage, skin, and vascular replacements are scrutinized and discussed. Finally, future challenges for improving PLLA-based scaffolding are suggested for new research.

## 2. PLLA as a Biomaterial

Tissue engineering applications of biomaterials have widely focused on studying synthetic polymers due to their interesting mechanical strength, elasticity, and biodegradability [9]. Recently, PLLA has been the midpoint in most cases of interest to tissue engineering for the fabrication of pure or hybrid scaffolds exhibiting high performance in terms of tissue regeneration [18,43].

### 2.1. Structure of PLLA

**Figure 1:**
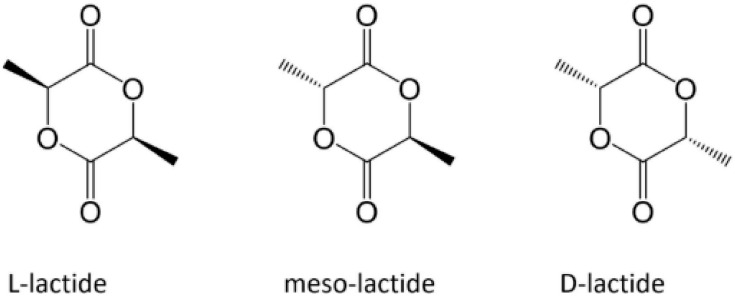
Enantiomeric forms of lactic acid [36]. Reprinted with permission from Frontiers.

PLLA is a homopolymer of the PLA family, which also includes the homopolymer PDLA and the copolymer PDLLA (Figure 1) [36]. The attractive properties of this family have motivated researchers to focus their study on these polymers owing to their non-toxicity and excellent physical and mechanical properties [44]. Based on its compositional and structural properties, PLA is a thermoplastic aliphatic polyester. It may include stereochemical forms that give specific properties to materials. For instance, PLLA and PDLA are semicrystalline polymers, whereas PDLLA is usually amorphous [15].

PLLA represents most PLA commercial grades. They are eco-friendly polymers obtained from 100% natural resources, such as cornstarch and sugar cane [45]. Its environmental advantage is linked both to the synthesis from non-petroleum-based processes (i.e., fermentation processes of oils, carbohydrates, or plants) [33] and the possibility of using eco-sustainable catalysts, such as cerium trichloride heptahydrate [46] and sorbitol [47]. PLLA production presents the advantage of lower energy use and, subsequently, lower costs [16]. PLLA is constituted by the cyclic dimer lactide LL- and has a crystalline structure between 30 and 40% [48] that can have several forms (α, α’, β, and γ); the α form is the most stable owing to its pseudo-orthorhombic unit cell, with the (10/3) chain adopting a helical conformation [45]. As a result, there is no symmetry on each side, leading to a distortion of the chain packing in the crystal lattice and energetical stability to the arranged chains [16].

### 2.2. Biological Properties of PLLA

PLLA is an FDA-approved polymer known for its low toxicity compared to other synthetic polymers [36]. The anti-infective effects of PLLA had been established during in vivo and in vitro experiments, facilitating the repair of the infected tissue [33,49]. One of the significant advantages of PLLA with respect to other biopolymers relies on the performance of PLLA during implantation ensuring adequate mechanical properties for prolonged regenerative processes [43]. However, researchers have advised some doubts about the hydrophobic surface of this polymer that might compromise its biocompatibility since it affects the amount of absorbed proteins and the cell adhesion [23,50,51].

Another crucial parameter that must be taken into account when determining the biological properties of one material is its biodegradation by-products that can cause local or systemic toxicity after implantation [43,52]. During PLLA degradation via hydrolysis, the polymer forms lactic acid as a by-product, typically present in the body and excreted as water and carbon dioxide [36]. The optimal degradation rate of a 3D support should match that of the ECM deposition of a specific tissue [6]. The kinetics of PLLA degradation depends on its crystallinity, strain, and microstructure that follows its deployment [10]. Lower crystallinity and higher strains lead to a faster degradation rate. PLLA is described as a resorbable synthetic polymer with slow degradation kinetics. This feature is given by the extra methyl group, leading to increased hydrophobicity and stability against hydrolysis [53]. The degradation time of PLLA is about 40 and 30 weeks in vitro [54] and in vivo [55], respectively.

### 2.3. Mechanical and Physical Properties of PLLA

PLLA is widely explored in tissue engineering applications because it is characterized by tunable mechanical properties [56]. Scaffolds fabricated from this bioresorbable material usually exhibit higher tensile strength (60–70 MPa) and modulus (2–4 Gpa) but lower elongation at break (2–6%) compared to other synthetic polymers, such as PCL and PDLA [53]. High-strength tissues, such as bone [50], ligaments [57], and dermis [58], have been extensively investigated by PLLA-based scaffolds to provide physical support during tissue healing. However, the mechanical behavior of PLLA strongly depends on its molecular weight, crystallinity, and aging characteristics [12].

PLLA is a thermoplastic polymer with thermal plasticity and transparency [59]. The latter depends crucially on the crystal morphologies and crystallinity [60]. PLLA has a melting temperature (T_m_) between 170 and 180 °C and a glass transition temperature (T_g_) of about 60 °C. T_m_ may vary due to impurities [45]. Moreover, it is characterized by a mechanical strength of about 4.8 GPa, depending on its molecular weight [15,36]. Due to its polyorthoester trait, PLLA presents a hydrophobic nature allowing it to degrade while remaining structurally intact [61]. Thermal degradation strongly reduces the length of PLLA chains, leading to a decrease in the molecular weight and structural stability under thermal and mechanical stresses [11]. The α-crystalline form of PLLA is the most thermodynamically stable owing to the C=O dipoles that are randomly oriented along the main chain, resulting in a non-polar form [62].

### 2.4. Reinforced PLLA-Based Biomaterials

**Figure 2:**
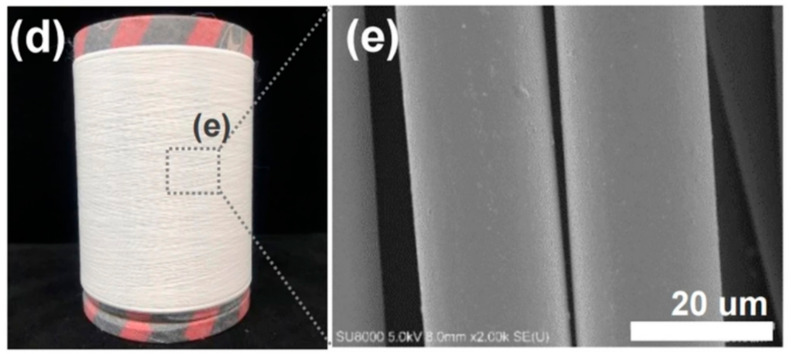
(**d**) Obtained as-spun PLLA fiber reinforced with BaTiO_3_ particles by the pilot-scale melt-spinning, and (**e**) FE-SEM image of the as-spun PLLA/BaTiO_3_ fibers. Reprinted with permission from Nature Publishing Group [64].

Reinforcement additives that modify a polymeric material can act as a trigger for material degradation and can improve mechanical properties [45,63]. Reinforced PLLA structures offer a potential advantage to improve scaffold properties. Mariano et al. [49] obtained and characterized a PLLA-based nancomposite reinforced with cellulose nanocrystals. According to their results, the presence of the filler increased the resistance of the melt flow and the Young’s modulus value. Similarly, Mg(OH)_2_ was used to improve the mechanical properties of PLLA-based composites [59]. Another material that can be added to obtain high-performance PLLA-based material is BaTiO_3_, which is specifically used to give piezoelectric properties to the polyester [64]. Figure 2 shows the global and microscopic structure of PLLA fibers reinforced with BaTiO_3_ particles fabricated by Oh et al.

Other additives have also been used to improve cell proliferation and protein absorption. Among them, chitosan [51] and hydroxyapatite [63] have been shown to enhance the osteoactivity of PLLA-based constructs. Moreover, PLLA was found to be itself a reinforcer for other materials. Natural polymers often used in the tissue engineering field lack good mechanical properties. Collagen and gelatin sponges [65] and silk fibers [66] resulted in higher strength and elastic modulus when reinforced with PLLA.

### 2.5. Drug/Growth-Factor-Loaded PLLA Systems

**Figure 3:**
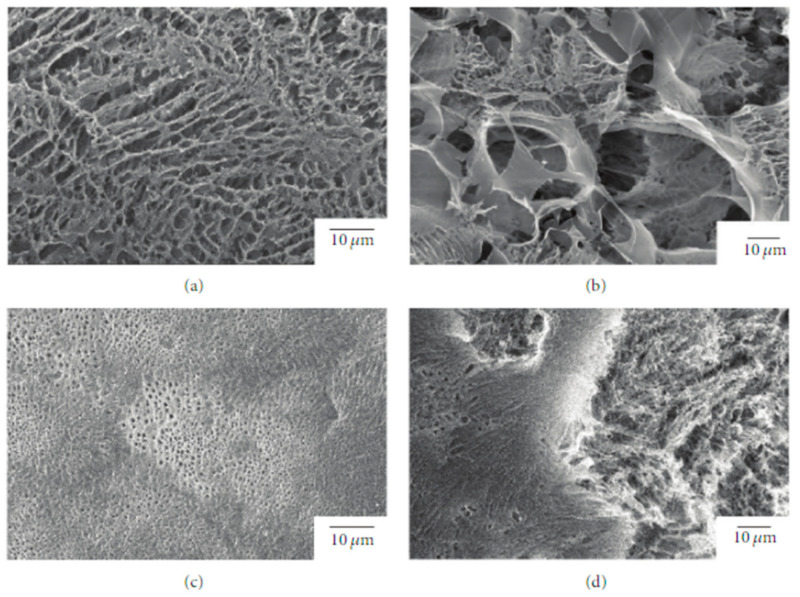
SEM images of the surface and interior of PLLA/rifampicin blend particles prepared by the drop freeze-drying method. (**a**) PLLA = 2.0 wt%, surface, (**b**) PLLA = 2.0 wt%, interior, (**c**) PLLA = 4.9 wt%, surface, and (**d**) PLLA = 4.9 wt%, interior. Reprinted with permission from Hindawi Limited.

Due to its relatively low biodegradation rate, PLLA is a suitable candidate for a prolonged drug delivery system [67]. In this regard, Sasaki et al. [68] developed a new drug-carrier material as blend particles composed of PLLA and rifampicin, an antibiotic drug used as an antibacterial and antifungal agent. The freeze-drying technique was used to prepare blend particles, starting from a solution composed of PLLA, rifampicin, and 1,4- dioxane. To avoid the heterogeneous adsorption method, the authors considered rifampicin suitable for their study because it is soluble in 1,4-dioxane, as well as PLLA. The obtained particles had an average porosity of 92 ± 3% and a specific surface area of 10–40 m^2^ g^−1^ (Figure 3).

The release kinetics of rifampicin in water were studied, resulting dependent on the morphology of the mixture particles that can be tuned by changing the concentration of the original solution and the freezing processing parameters.

Cao et al. evaluated the ability of the PLLA/polyhydroxybutyrate (PHB) for drug release [39]. They prepared the scaffold using solvent-free melt electrospinning and different concentrations of the drug dipyridamole (DPD). The authors found that DPD acts as a plasticizer for PLLA, thus allowing them to carry out the melting electrospinning process at a lower temperature if compared to neat PLLA. From their findings, the fibers containing DPD showed a rougher surface and nonuniform diameters within a single fiber than the fibers without DPD. The release profile of DPD was studied at different PLLA/PHB ratios (9/1 and 7/3) with the same drug concentration (1%), showing that the 9:1 PLLA/PHB system was more resistant to polymer hydrolysis than the 7:3 one. In line with this result, the rate of diffusion transport was approximately two times higher for the 7:3 PLLA/PHB fibers than for the 9:1 PLLA/PHB fibers. Overall, the authors proved the ability of this hybrid system to enhance sustained drug release.

In a more recent study, Guidotti et al. [69] developed a novel A-B-A triblock copolymer, based on PLLA as the A block, to prepare micro and nanoparticles for controlled drug delivery. Block B was a poly(butylene/triethylene succinate) P(BSTES) copolymer system in which the hydrophilic/hydrophobic ratio and hydrolytic degradation kinetics were adjusted for an adequate release of the drug by controlling the block length. To investigate the application of these new materials for manufacturing micro and nanoparticles for controlled drug delivery, the effect of both chemical structure and molecular architecture as well as of particles size was evaluated on drug release kinetics. Specifically, the authors loaded the micro and nanoparticles with dexamethasone, a synthetic corticosteroid with rapid gastrointestinal absorption. From their results, after observing the drug release from the copolymer and pure PLLA after 8 h of incubation under physiological conditions, the drug release from the copolymers was complete, while that from the PLLA matrix still continued, although more than 80% of the drug had already been released during incubation. The authors suggested that, in copolymer microparticles, the drug was released through a diffusion-controlled mechanism. Instead, the release of the drug from the PLLA microparticles was also affected by the erosion process that occurs in the subsequent phases.

**Figure 4:**
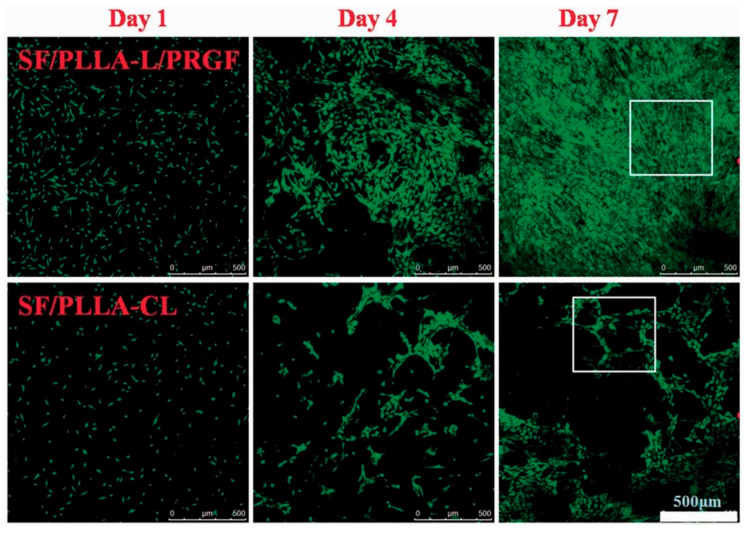
Confocal laser images of SMCs grown on SF/PLLA-CL/PRGF and SF/PLLA-CL for 1, 4, and 7 days. Reprinted with permission from Oxford Academic [73].

In the TE field, growth factors (GF) are a fundamental element since they have the potency to induce and enhance cellular responses [18,70]. According to Shen et al. [20], tissue regeneration with only cells and scaffolds is often unsuccessful, and exogenous growth factors must be in place to initiate the regeneration process. In this context, PLLA-base scaffolds incorporating growth factors were proposed to improve their biological activity and induce cell differentiation [38,70,71,72]. Yin et al. [73] fabricated silk fibroin/poly (L-lactide-e-caprolactone) (SF/PLLA-CL) vascular graft loaded with platelet-rich growth factor (PRGF) by the electrospinning technique. PRGF was incorporated to promote effective smooth muscle cell (SMC) growth and infiltration into the graft. From their findings, SF/PLLA-CL/PRGF possessed a slightly higher strain (282%) and lower elastic modulus (1.8 MPa) than SF/PLLA-CL (259% and 2.5 MP, respectively), showing appropriate mechanical properties compared to native blood vessels. Concerning the cellular behavior, PRGF enhanced cellular infiltration into the graft to a profound extent and induced fast SMC growth (Figure 4).

In cartilage and bone TE, Zhang et al. [74] introduced poly(hydroxyethyl) methacrylate (PHEMA) blocks to conjugate GF-mimicking peptides on PLLA scaffold surface and fabricate a scaffold with improved bioactivity. Two different GF-mimicking peptides were separately conjugated, i.e., TGF-β1 and BMP-2 for inducing chondrogenic and osteogenic differentiation of BMSCs, respectively. These peptides were introduced into the PLLA scaffold after adjusting the density of acrylic groups in the copolymers during the molecular synthesis to conjugate more peptides. Based on the reported in vitro and in vivo tests, incorporating GF-mimicking peptides in PLLA–PHEMA scaffolds actively directed the stem cells along their respective differentiation pathways. They also promoted cartilage and mineralization compared to the control unconjugated scaffolds.

## 3. PLLA-Based Scaffold Processing for Tissue Engineering

The scaffold material plays a crucial role in providing functional support to enhance extracellular matrix deposition and cellular growth while temporarily replacing the mechanical function of a living tissue [75]. In literature, many research works have focused on PLLA, either as pure material or composite architecture, for preparing porous structures employed as TE scaffolds [18,76,77,78,79].

**Figure 5:**
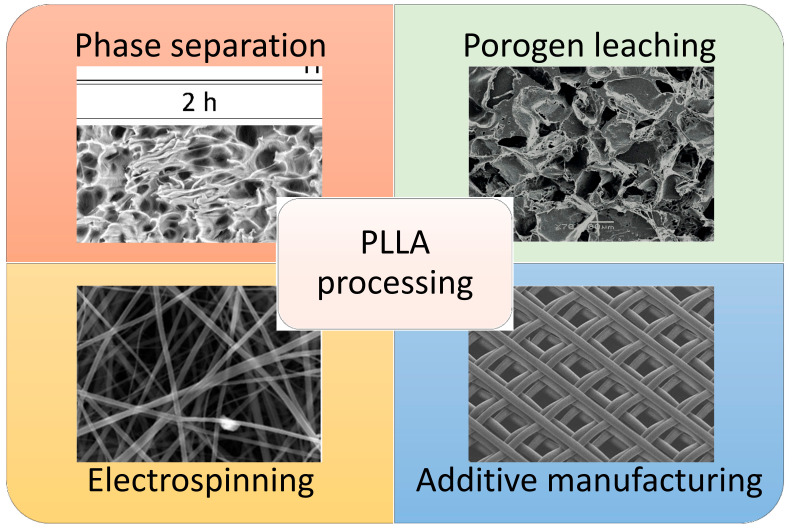
Pore morphologies of PLLA-based scaffolds prepared with some of the most used processing techniques. Reprinted with permission from MDPI [80,81], Hindawi [81], and Elsevier [82].

As schematized in Figure 5, several different approaches can be adopted for the fabrication of PLLA-based scaffolds resulting in specific pore morphologies.

Table 1 reports the main fabrication approaches that may be adopted for PLLA-based scaffold fabrication along with their processing parameters, main properties, advantages, and disadvantages. TIPS, DIPS, additive manufacturing, and porogen leaching approaches allow one to obtain three-dimensional PLLA-based scaffolds with relatively high mechanical properties that meet the requirements of hard tissues such as bone [24,63,83]. On the other hand, PLLA-based scaffolds produced via electrospinning are usually bi-dimensionally shaped or eventually wrapped in cylindrical collectors to form vessel-like structures. For this reason, combined with the relatively low elastic modulus of these systems, PLLA-based electrospun scaffolds are mainly engineered for skin or blood vessel regeneration [65,84,85]. Despite the need for organic solvents in most of the solution-based processing proposed in literature, several articles ensure the achievement of a final structure without any remaining solvent trace when investigated, hence avoiding cytotoxicity [86,87].

## 4. Pure PLLA Scaffold

PLLA is a biodegradable, FDA-approved polymer. Pure PLLA scaffold is considered suitable for tissue engineering purposes due to its tunable mechanical properties, i.e., a 0.1 kPa–80 MPa compressive modulus dependent on the structure, pore size, and porosity (Table 1). These properties make PLLA a feasible material for being fabricated through several techniques (such as additive manufacturing [121], electrospinning [122], phase separation [123], and particulate leaching [124]) as adequate support for tissue regeneration. Pisanti et al. [125] produced PLLA scaffolds using either supercritical carbon dioxide (SC-CO_2_) gel drying and combining this technique with porogen leaching, thus obtaining nanoporous and microporous structures, respectively. Their research showed that the produced scaffolds with the largest pore size induced a higher proliferation of human mesenchymal stem cells (hMCSs). La Carrubba et al. [83,88,91,126,127] have extensively studied PLLA scaffolds produced via phase separation techniques. While investigating both TIPS and DIPS approaches, they optimized operating parameters to obtain porous matrices with different porosities and pore sizes, adequate to host various types of cells. The TIPS technique has also been combined with the sugar template method to produce PLLA matrices while precisely regulating their porous architecture [96]. Another central feature of PLLA scaffolds produced by phase separation is their capability to control their biodegradability [128].

Electrospinning is an alternative technique to produce pure PLLA scaffolds for tissue engineering [62,129,130]. Electrospun PLLA has a nanofibrous structure whose porosity and thickness depend on the initial concentration of the polymer solution [9]. The electrospinning process can fabricate PLLA scaffolds for sensing applications, where piezoelectric properties are required for signal monitoring and detecting dynamic tactile stimuli [62]. By stretching the randomly orientated chains of PLLA in the thermodynamically stable α-crystalline form, the electrospinning process can transform the latter form into the β-crystalline form, where molecular chains are aligned along the stretched direction, thus inducing polarization and piezoelectricity.

Researchers have shown that it is possible to fabricate 3D scaffolds from PLLA powder through the rapid prototype technique [61]. However, 3D-printed PLLA scaffolds seemed to exhibit large-sized pores that cannot support cell adhesion [128,131].

### Surface Modifications

Although pure PLLA scaffolds have offered excellent results, their hydrophobic nature limits the cell–material interactions and the biological recognition on the material surface. Surface treatments can improve the surface bioactivity of PLLA, thus providing an ideal environment for promoting cell adhesion [61]. The deposition of bioactive molecules is the most commonly used method for surface treatment. Hydroxyapatite (HA), chitosan, and collagen have been used as coatings to improve the biological properties of PLLA-based scaffolds [15]. Kabiri et al. [132] incorporated single-walled carbon nanotube (SWCNT) within an electrospun PLLA scaffold. Their research showed that such inclusion endows the aligned nano-sized fiber substrate with conductivity, promoting nerve regeneration. In the work of Ahmadi et al., Wharton’s jelly (WJ), a tissue that surrounds the umbilical cord vessels, was used as a coating for PLLA electrospun nanofibers [129]. According to the authors, coating PLLA nanofibers with WJ has multiple benefits for tissue-engineered scaffolds because WJ is rich in collagen, fibrous and interstitial proteins, and signaling molecules. These compounds could facilitate cellular attachment, proliferation, infiltration, and differentiation. Indeed, WJ-PLLA scaffolds showed higher proliferation of human mesenchymal stem cells (MSCs) than tissue culture plate (TCP) and PLLA scaffolds. Nevertheless, the surface properties of the PLLA scaffold, such as porosity and fiber diameter, were not significantly affected by WJ coating.

**Figure 6:**
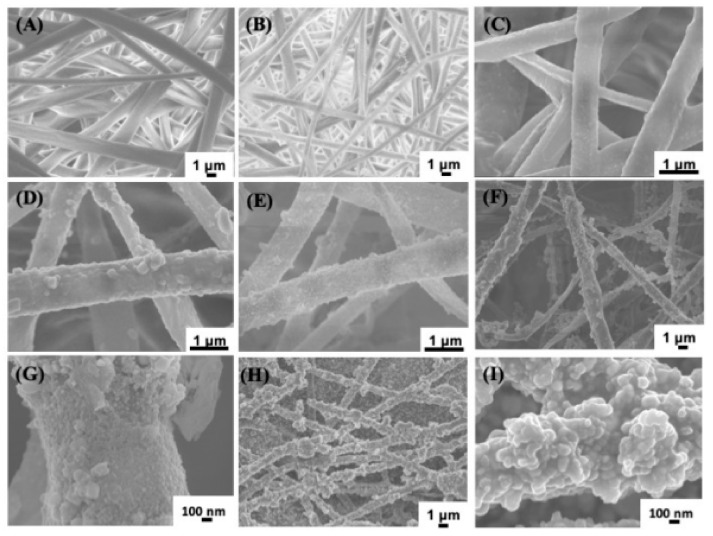
SEM images of different samples. Poly(L-lactide) (PLLA) (**A**), PLLA@PDA (**B**), PLLA@Ag1 (**C**), PLLA@Ag3 (**D**), PLLA@Ag6 (**E**), PLLA@Ag9 (**F**,**G**), and PLLA@Ag 24 (**H**,**I**). Reprinted with permission from Frontiers [130].

In a recent study, silver nanoparticle (AgNP)-coated PLLA membranes (PLLA@Ag) were produced (Figure 6) in order to impart antibacterial properties to the PLLA electrospun scaffold [130].

The modified PLLA membranes displayed superior antibacterial properties than pure PLLA, showing great potential for clinic application.

Another technique commonly used to modify the polyester surface is the plasma treatment. This method allows for improving the hydrophilicity of the polymer without affecting its bulk properties [15]. Liu et al. [133] applied oxygen plasma treatments on PLLA scaffolds fabricated by electrospinning. According to their findings, plasma treatments reduced the polymer contact angle and increased the surface free energy, resulting in changes in the polar components that made PLLA more hydrophilic. Therefore, during plasma treatments, the operating parameters, such as plasma power and treatment time, should be appropriately set to avoid changes in the biodegradability properties of the polymer [134].

## 5. PLLA Hybrid Scaffolds

**Figure 7:**
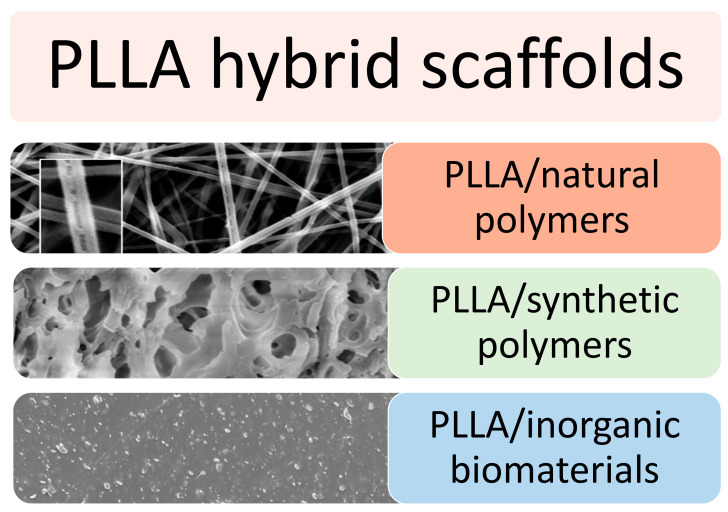
Microstructure of PLLA-based hybrid scaffold in combination with natural polymers, synthetic polymers or inorganic biomaterials. Reprinted with permission from RSC [135] and MDPI [136,137].

Although surface modifications exert a positive effect on cell adhesion and cell interaction, they can alter the chemistry of the surface. This phenomenon can cause excessive protein absorption and unwanted chemical reactions, thus affecting the in vivo response. Hence, hybrid scaffolds have improved the properties of the pure biomaterial while overcoming the issues related to surface modifications. Specifically, PLLA has been blended with natural and synthetic polymers and ceramic biomolecules to meet the desired physical, mechanical, and biological properties. In addition, some biopolymers may offer adhesion sites for cells and thus may allow for a better mimicking of the native ECM (see Figure 7).

### 5.1. PLLA/Natural Polymers Hybrid Scaffolds

Natural polymers have been widely used in blends with synthetic polymers since their ECM-like structure enhances the cell growth and adhesion on the hybrid construct. There are several lines of research for tissue engineering applications aiming to fabricate hybrid scaffolds from PLLA and natural polymers, such as collagen [22], chitosan [51,113], silk fibroin [66,109], and gelatin [107,138].

Gelatin is a collagen-derived molecule that easily entangles into coils in aqueous solutions thanks to covalent crosslinks. Yazdanpanah et al. [84] showed that PLLA/gelatin scaffolds revealed good mechanical properties and excellent biological characteristics. Their graded scaffolds present PLLA in the core and gelatin in the surface, thus preserving PLLA mechanical strength and enhancing the biological affinity, thanks to gelatin molecules in the structure. Compared to collagen, gelatin is more soluble in water and provides lower antigenic and immunogenic responses in physiological conditions [139], thus better supporting the differentiation of various cell lineages. On the other hand, collagen-based, hybrid PLLA scaffolds seemed to promote cell attachment and proliferation more than PLLA-gelatin hybrid scaffolds, as observed by Lu et al. [65]. As a fact, gelatin is partially denatured collagen, resulting in lower bioactivity. However, collagen-based scaffolds, like gelatin-based hybrid scaffolds, produced larger surface areas of regenerated skin with respect to the collagen control group after in vivo subcutaneous implantations.

Recently, the suitability of polysaccharides for cell attachment has been exploited to improve the biological characteristics of synthetic polymers [110,114]. Among these natural materials, chitosan, a natural polysaccharide widely used in the TE field, showed unique anti-bacterial properties. In the paper by Zen et al. [111], collagen and chitosan were used to enhance the cell viability of PLLA scaffolds. PLLA–chitosan–collagen scaffolds were fabricated by electrospinning at various concentrations of chitosan (i.e., 0.5% and 0.6% dissolved in a 10% PLLA and 1% collagen solution). These scaffolds were found to be suitable for mimicking blood vessels because of their high hemocompatibility (all materials had hemolysis percentages lower than 5%, declared safe for direct contact with blood) and a burst pressure between 1371 and 2593 mmHg, in line with the human arterial burst pressure (1264–3196 mmHg). By increasing the chitosan percentage, the tensile strength of the hybrid scaffolds increased, thus meeting the standards of mechanical properties of vascular graft materials.

Silk fibroin (SF) is a natural protein that can be synthesized starting from various species of worms [140]. Silk fibroin can be combined with synthetic polymers to increase their cell affinity and adhesion [1,19,141]. Research has shown that silk fibroin could improve the hydrophobic behavior of PLLA by preparing electrospun SF/PLLA scaffolds [109]. This research demonstrated that fibrous SF/PLLA constructs had excellent spreading, ECM secretion of chondrocytes, and tunable degradation profile, thus representing a promising material for cartilage TE.

### 5.2. PLLA/Synthetic Polymer Hybrid Scaffolds

The major limitation of using natural polymers is their low mechanical strength and thermo-sensitivity, which also affects the process of scaffold fabrication, such as when using electrospinning [142] or additive manufacturing [141]. When 3D printing natural polymers, viscosity can fluctuate, making it hard to fabricate reproducible scaffolds with a precise structure. Natural polymers in an aqueous environment possess poor stability as they swell and collapse, causing a decrease in the interconnectivity between pores [142]. Compared to natural-based polymeric scaffolds, those developed with synthetic materials can be easily manufactured [19] since they present more tunable properties, such as molecular weight, crystallinity, and transition temperatures [21]. Recently, synthetic hybrid scaffolds were proposed in TE as materials with complete control over mechanical properties and architecture [86]. Scaffolds composed of PLLA and synthetic polymers, such as PCL [106,143], PLGA [103,144], PLA [145,146], and PVA [147], have been fabricated for TE applications.

**Figure 8:**
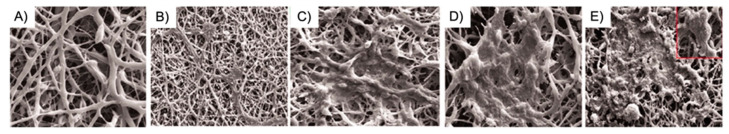
Three-dimensional electron micrograph images of (**A**) unseeded hybrid PLLA/PCL scaffolds, (**B**) hiPSCs-seeded scaffold on starting day, (**C**) induction cells to assembling, (**D**) induced cells to aggregate, and (**E**) islet-like clusters. Scale bars are (**A**): 10 mm; (**B**–**E**): 100 mm [106]. Reprinted with permission from Taylor & Francis.

The PCL is an aliphatic, semi-crystalline, and non-toxic polyester that is usually adopted to enhance the flexibility of the scaffold owing to its rubbery state [148]. Hybrid scaffolds made up of PLLA and PCL have been investigated for their versatile degradation rate, optimal porosity, and good resistance to high temperature and pressure [112]. Shamsah et al. [143] studied the in vitro degradation of 50:50 PCL/PLLA electrospun scaffolds for a period of up to six months. The crystallinity significantly increased over time, along with tensile strength and weight loss. The PCL/PLLA scaffolds revealed active hydrolysis and higher stiffness than pure PLLA and PCL scaffolds. Nanofibrous PCL/PLLA scaffolds were fabricated using electrospinning and examined to assess their feasibility for the differentiation of human-induced pluripotent stem cells (hiPSCs) to insulin-producing cells [106]. The in vitro study showed interesting cell/scaffolds interaction that resulted in the formation of islet-like clusters of differentiated cells (Figure 8).

PVA is an additional widely applied synthetic polymer in TE owing to its transparent nature, flexibility, and slow degradation kinetics [149]. Scaffolds composed of PLLA and PVA have been fabricated by the electrospinning technique, a simple and cost-effective method for successfully producing blended scaffolds. In a study by Mahboudi et al. [147], an electrospun PLLA/PVA scaffold was examined in terms of chondrogenic differentiation of human induced pluripotent stem cells (iPSCs). The hybrid scaffolds possessed fibers with uniform and smooth morphology and showed higher viability and proliferation rate of the hiPSCs than the 2D culture group. Moreover, the surface hydrophilicity of PVA/PLLA constructs can be optimized by applying oxygen-induced plasma treatment [149].

Hybrid PLLA/PLGA scaffolds showed promising applications in tissue engineering. PLGA is a copolymer of PGA and PLA, which is well-known for its tunable biodegradation that can be controlled by altering the ratio of PLA and PGA [108]. In a study carried out by Saito et al. [144], the effects on bone formation in vivo of pure PLLA and 50:50 PLLA/PLGA scaffolds were compared. Although the PLLA and 50:50 PLGA scaffolds had similarly defined pore sizes, porosities, and surface-to-volume ratios, the hybrid scaffolds resulted in faster degradation and higher bone ingrowth than pure PLLA scaffolds. On the other hand, the mechanical properties of PLLA scaffolds were more suitable for bone tissue than those of 50:50 PLGA scaffolds for the entire study period. Different microstructures of PLLA/PLGA scaffolds were fabricated by freeze-drying at different solution concentrations and were analyzed to see how their microarchitecture and mechanical properties affect the behavior of fibroblastic cell types [150]. The study showed that, when increasing polymer concentration, the compressive modulus of the scaffolds decreased and the tensile modulus increased, resulting in better fibroblast cell attachment and proliferation at lower concentrations of the polymeric solution.

Scaffolds with PLA and PLLA blends were studied deeply by Carfì et al. [151,152,153]. In these studies, scaffolds with different PLLA/PLA proportions were produced by phase separation techniques and tested as functional porous scaffolds. The in vitro degradation rate, pore morphology, and crystallinity were evaluated for the designed scaffolds with and without seeded cells. Their results showed that PLLA/PLA hybrid scaffolds had a faster degradation rate than pure PLLA and a lower mechanical strength at a higher PLA percentage.

Moreover, PLLA has been combined with more than one polymer to obtain optimal structures that are suitable for specific TE applications. Among them, PLLA/chitosan/collagen constructs [111], PLGA/PLLA/PDLLA fibers [103], and PLLA-PEG-PLLA/PDLA blends [154] were characterized to assess their high performance as biodegradable materials.

### 5.3. PLLA/Inorganic Biomaterials Composite Scaffolds

Among inorganic biomaterials, ceramic materials have been used in tissue engineering applications thanks to their high resilience and suitability for mineralized tissues, such as bone [128]. When ceramics are processed alone, they cannot be easily transformed into interconnected porous structures due to their brittle nature [61]. By combining ceramics and polymers, scaffolds with mechanical properties matching those of a load-bearing tissue could be produced [155]. In the TE field, the most common bioceramics are hydroxyapatite, bioactive glasses, and calcium phosphate. They are bioactive, biocompatible materials that have been used as fillers for bone defect repair [156]. Therefore, ceramic materials can be combined with polymers, as they act either as reinforcing agents and/or biomimetic cues to guide the differentiation of cells and shorten the time required for mineralization [63]. Among synthetic polymers, PLLA has been successfully used to fabricate composite polymer/ceramic scaffolds with controlled macro and microstructures.

**Figure 9:**
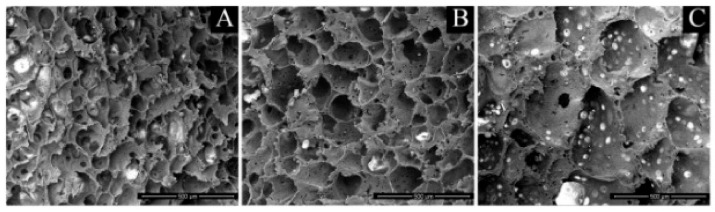
SEM micrographs of the scaffold prepared with a 90/10 PLLA/HA ratio at different demixing temperatures, keeping the demixing time constant. (**A**) 25 °C; (**B**) 30 °C; (**C**) 35 °C. Reprinted with permission from Elsevier [97].

Hydroxyapatite (HA) is an osteoconductive mineral similar to the inorganic component of the native bone [157]. The non-toxicity of HA suggests its use for coating the hard tissue and metal implant [108]. This bioceramic can be combined with PLLA to form a composite scaffold with optimum bone-like properties. A PLLA/HA construct with an integrated structure fabricated by the solvent casting technique [102] exhibited better thermal stability and higher decomposition temperature than the neat polymer. The high stiffness of HA maintained the mechanical stability of the composite structure at higher temperatures than pure PLLA. In a study made by Vitrano et al. [158], PLLA/HA scaffolds (at 10% and 20% of HA content) produced via TIPS were examined for bone tissue implantation. The morphological investigations demonstrated a homogeneous HA distribution, thus illustrating that, during the TIPS process, the sedimentation of HA particles does not occur even at high HA concentrations. The in vitro cell culture with MC3T3-E1 pre-osteoblastic cells on composite PLLA/HA scaffolds was carried out by Carfì et al. [97]. In their research, composite scaffolds were fabricated by the TIPS technique either at different PLLA/HA ratios or demixing temperatures (Figure 9).

According to their findings, HA did not influence the morphology of the scaffolds and the number of viable cells. However, PLLA/HA scaffolds exhibited higher ALP activity than pure PLLA structures at 21 and 27 days.

Bioglass (BG), or bioactive glass, is a biocompatible calcium phosphate variant that is used in bone therapies to increase the rate of bone-like tissue formation [149]. When using Bioglass, the growth of scar tissue is rare when tissue bonds, thanks to the high rate of surface reactions of this material [159]. Bioglass increases osteoconductivity and biological properties of the scaffold by enhancing cell adhesion and the proliferation of osteoblasts on the surface of the scaffolds [89]. In recent years, a 45S5 bioactive glass (composition in wt%: 45% SiO_2_, 24.5% Na_2_O, 24.4% CaO, and 6% P_2_O_5_) has gained considerable interest as a coating for polymeric scaffolds to stimulate new bone formation both in vivo and in vitro. In cell culture experiments, the results revealed that BG/PLLA composite scaffolds enhanced the alkaline phosphatase (ALP) activity of MC3T3-E1 cells and osteoconductive gene expression with a content-dependent behavior [24,89]. BG has been demonstrated to be beneficial for the significant improvement of the osteogenesis of composite scaffolds during in vivo animal experiments [15,24].

**Figure 10:**
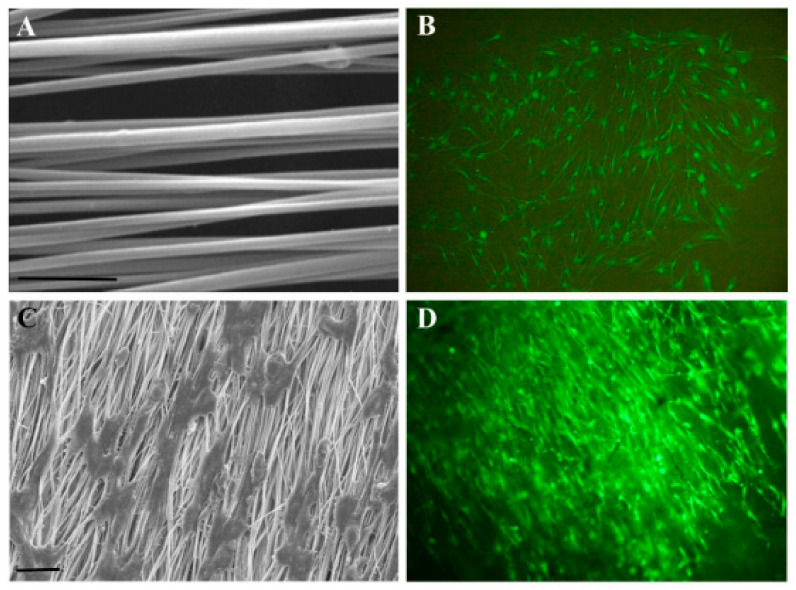
Effect of guidance cues on the alignment of OEC. (**A**) Aligned SWCNT/PLLA nanofibers used as the substratum for OEC, scale bar = 10 µm; (**B**) OEC grown on culture plates showing random orientation, magnification 100×; (**C**) SEM micrographs of OEC aligned on nanofiber SWCNT/PLLA scaffolds, scale bar = 2 µm; (**D**) Fluorescence image of aligned OEC grown on SWCNT/PLLA nanofibrous scaffolds, magnification 100×. Reprinted with permission from EXCLI Journal [130].

Besides ceramics, other inorganic materials have been used as matrix filler to produce composite PLLA-scaffolds, albeit to a lesser extent. Recently, Kang et al. [160] attempted to add magnesium hydroxide nanoparticles (nMH) to the PLLA matrix as a bioactive filler that can suppress inflammatory responses by neutralizing the acidified environment caused by the degradation of PLLA. From their findings, the incorporation of nMH enhanced mechanical properties (such as Young’s modulus) and reduced bulk erosion during hydrolytic degradation, leading to lower cytotoxicity and immunogenicity. According to the authors, nMH has great potential as an additive to improve the mechanical and biological properties of biodegradable polymers used in various biomedical applications, especially vascular stents and orthopedic implants. In another work, Kabiri et al. [130] aimed at fabricating a conductive aligned nanofibrous substrate for nerve tissue engineering purposes. Hence, they incorporated single-walled carbon nanotubes into PLLA nanofibrous scaffolds and then assessed their cytocompatibility with olfactory ensheathing glial cells (OEC), used to treat nerve injuries. Under the hypothesis that functionalizing the PLLA nanofibers with an electrically conductive compound could aid in mimicking the conductive nature of the nerve tissues, the authors successfully demonstrated that OEC adhered and proliferated well on these scaffolds and get aligned along the direction of the fibers (Figure 10). Overall, the SWCNT/PLLA scaffold resulted in a potential construct to promote axonal outgrowth and glial migration from the nerve into the graft, thus improving nerve regeneration.

## 6. Applications of PLLA-Based Scaffold in Tissue Engineering

**Figure 11:**
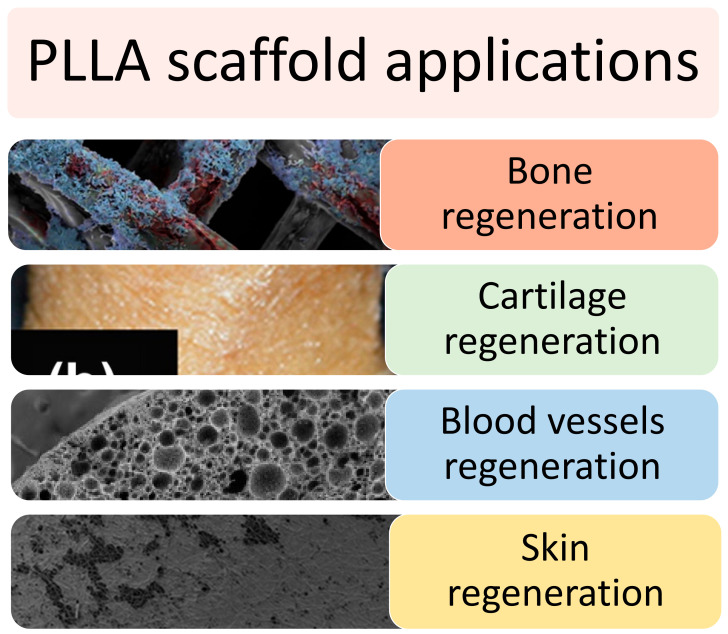
Structure of PLLA-based scaffold designed for bone, cartilage, blood vessel, and skin regeneration. Reprinted with permission from Elsevier [82], Taylor & Francis [113], Dovepress [161], and RSC [162].

PLLA is one of the first synthetic polymers that has been recognized as an attractive material for tissue engineering. In this context, PLLA properties have been tailored to produce PLLA-based scaffolds for specific tissue engineering applications including bone, cartilage, blood vessels, and skin tissue regeneration, as schematized in Figure 11. Table 2 summarizes the material combination, the processing, and the properties of PLLA-based scaffolds designed for specific tissues.

### 6.1. Bone Tissue

Bone tissue is a dynamic and complex organization of blood vessels and different active cells, including osteoblasts, osteoclasts, and osteogenic cells. Depending on the microbiologic conditions, osteogenic cells, which are undifferentiated, may become osteoblasts or osteoclasts. In this way, they actively regulate bone homeostasis [119,149,163]. Bone is a self-healing tissue when a small defect occurs. However, treating critical defects, such as pathological fractures and complex breaks, is still a big challenge [164,165]. PLLA-based scaffolds could provide functional support for bone tissue repair.

PLLA-based scaffolds fabricated by phase separation techniques were widely studied in vitro as potential matrices for bone regeneration by Ciapetti et al. [63]. They analyzed the morphological, biochemical, and gene expression of human MCSs seeded on PLLA-based scaffolds, including single-walled carbon nanotubes, micro-hydroxyapatite particles (HA), and bone morphogenetic protein 2 (BMP2) molecules. Their findings revealed that the addition of HA and BMP2 to the composite enhanced the number of cells on the scaffolds and the collagen production during the mineralization phase, respectively. On the other hand, the SWCNT-added PLLA scaffold exhibited a low osteoconductive ability. Recently, composite PLLA/HA foams produced by TIPS with different hydroxyapatite contents (10, 25, 50, 75, 90 wt.% of the HA) were analyzed deeply for bone TE [115]. This research revealed that the compressive properties and proliferation rate of osteoblast cells were proportional to the HA content of the foam, reaching optimal properties in PLLA/HA 25/75 scaffolds. In vivo studies using PLLA-based scaffolds fabricated by the phase separation technique indicated their suitability for bone regeneration. Weng et al. [92] compared the in vivo regeneration of a 15 mm ulna bone defect in a rabbit using PLLA and PLLA/PCL scaffolds. They found a slow degradation rate and low bone mineral density for both PLLA and PLLA/PCL scaffolds, whereas the callus formation of PLLA/PCL was better than for PLLA. As a matter of fact, PCL does not produce acidic degradation products, hence counteracting the disadvantages of PLLA.

In bone TE, electrospinning has been widely used to produce nanofibrous scaffolds with a structure close to the nanoscale collagen fibers of bone [166]. Electrospun PLLA scaffolds were produced using a surface modification to direct cellular differentiation to the bone lineage and achieve optimal bone regenerative performances. In a recent study by Fu et al. [167], electrospun PLLA nanofibers were successfully modified by the surface deposition of osteogenic ECM (secreted by MC3T3-E1 cells). Then, these scaffolds were used to examine the mouse bone marrow stromal cells (mBMSCs) response on seeded scaffolds. The results indicated that mineral growth, ALP activity, and cell morphology were at optimum conditions in the modified constructs if compared to pure PLLA nanofibers.

Electrospun PLLA/gelatin nanofiber scaffolds were even examined for in vivo bone formation using a critical-size rat calvarial defect model [168]. In this direction, the incorporation of hydroxyapatite into nanofibers was analyzed in terms of the increased osteoinductivity of the scaffolds.

After implantation, PLLA/gelatin scaffolds showed marginal ossification than PLLA/gelatin/HA scaffolds, which induced faster bone regeneration during the first six weeks. However, no significant differences between the two types of scaffolds were observed after ten weeks of the in vivo experiment.

Functional PLLA-based scaffolds to promote bone tissue repair have also been produced by additive manufacturing technologies, specifically in combination with bioceramic materials. Since the major limitation of bioceramics is their inability to fuse in the presence of thermoplastic polymers [118], low-temperature deposition manufacturing (LDM) has been developed to produce PLLA/ceramics composite scaffolds [18,118,120]. All these scaffolds exhibited high porosity and mechanical strength close to those of spongy human bone, supporting bone-like cell proliferation and in vivo bone conductivity.

### 6.2. Cartilage Tissue

Cartilage is a load-bearing connective tissue containing chondrocytes as cells and a surrounding extracellular matrix, which is a complex network of water, collagen, proteoglycans, and other noncollagenous proteins [169]. Progressive aging and injury can cause cartilage damages, thus leading to different diseases, such as osteoarthritis. The self-repair capacity of cartilage is extremely limited because of the absence of progenitors cells and vascularization in the tissue [170]. Recent tissue-engineering biotechnologies have examined plenty of scaffold architectures and different MSC sources, as well as combinations of synthetic polymers and living cells, to build an implantable replacement for the regeneration of cartilage [156]. Synthetic polymers and high-modulus hydrogels are the most often used materials as scaffolds for tissue-engineered cartilage [78].

**Figure 12:**
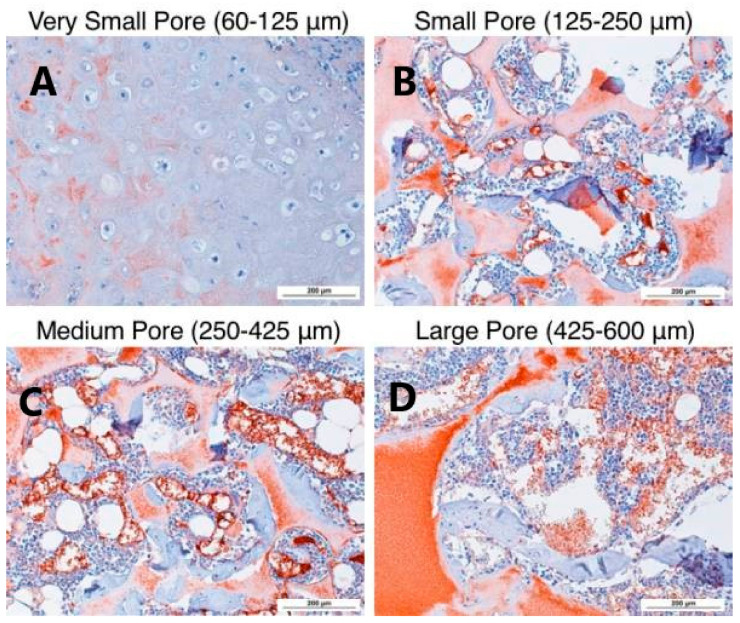
H&E histological analysis after 8 weeks of subcutaneous implantation at 100× magnification. Small pore scaffold (60–125 µm) (**A**) contained cartilage with typical morphology in the center of the scaffold. Small (125–250 µm), (**B**) medium (250–425 µm), (**C**) large-pore (425–600 µm), and (**D**) scaffold-supported bone formation on pore walls, shown by pink staining of bone matrix, with bone-marrow-like tissue within the pores. N = 3 for each group. Scale bars = 200 μm. Reprinted with permission from Acta Materialia Inc. [90].

For instance, PLLA nanofibrous scaffolds fabricated via phase separation combined with porogen leaching have been demonstrated to be excellent candidates for a wide variety of in vivo and in vitro cartilage repair strategies. This feature is due to the high porosity and interconnectivity of these scaffolds, as well as their good degradable properties [21]. Gupte et al. [90] prepared PLLA nanofibrous scaffolds with different pore sizes via the TIPS technique. Then, they studied the relationship between pore size and chondrogenesis both in vitro and in vivo (Figure 12).

The results show that scaffolds with a small pore size (125–250 µm) significantly induced the in vitro chondrogenic differentiation of human BMSCs and better supported the in vivo cartilage formation than large pore size (425–600 µm) constructs.

However, although nanofibrous PLLA-based scaffolds have shown beneficial effects on cartilage repair, their fibrous nature leads to limited load-bearing properties [140]. Highly porous scaffolds have been extensively studied in terms of mechanical properties and chondrogenesis support for cartilage TE. Recently, Rajzer et al. [107] fabricated a layered Gelatin/PLLA scaffold by electrospinning and 3D printing for nasal cartilage reconstruction. Fabricated scaffolds had nanofibrous gelatin membranes on the surface of porous 3D printed PLLA scaffolds. The authors tested the influence of the internal architecture of the porous 3D printed scaffolds on their mechanical properties, resulting in a maximum tensile strength of 18 MPa, in line with the range of 0.8–25 MPa that commonly occurs for cartilage tissue [171]. The mechanical properties of PLLA-based, sponge-like scaffolds for cartilage TE were also investigated by Mallick et al. [113]. They fabricated hybrid chitosan/PLLA scaffolds at different concentrations by means of the freeze-drying method. From their findings, the mechanical properties of the scaffolds decreased as the proportion of PLLA increased; however, mechanical stability was reached when the ratio of chitosan:PLLA was 70:30, which also showed to be the most enhanced support for the proliferation and attachment of cells. The effect of porous PLLA scaffolds and their pore dimensions on the proliferation and differentiation of chondrocytes was also studied by Conoscenti et al. [83]. They produced highly porous scaffolds with controlled pore sizes by the TIPS technique. From a gene expression analysis, scaffolds with an average pore size of 100 µm seemed to promote higher expression of the chondrogenic genes than PLLA with a pore size of 200 µm. This aspect occurred both for articular cartilage and nasoseptal chondrocytes. Additionally, these scaffolds were tested for the chondrogenesis of MSCs. The results revealed a higher expression of cartilaginous genes in scaffolds with 100 µm-pore size.

All the research mentioned indicated that the adequate size of the scaffolding pores improves the functional properties of cartilage, thus providing an effective strategy for the regeneration and repair of this tissue.

### 6.3. Blood Vessels

**Figure 13:**
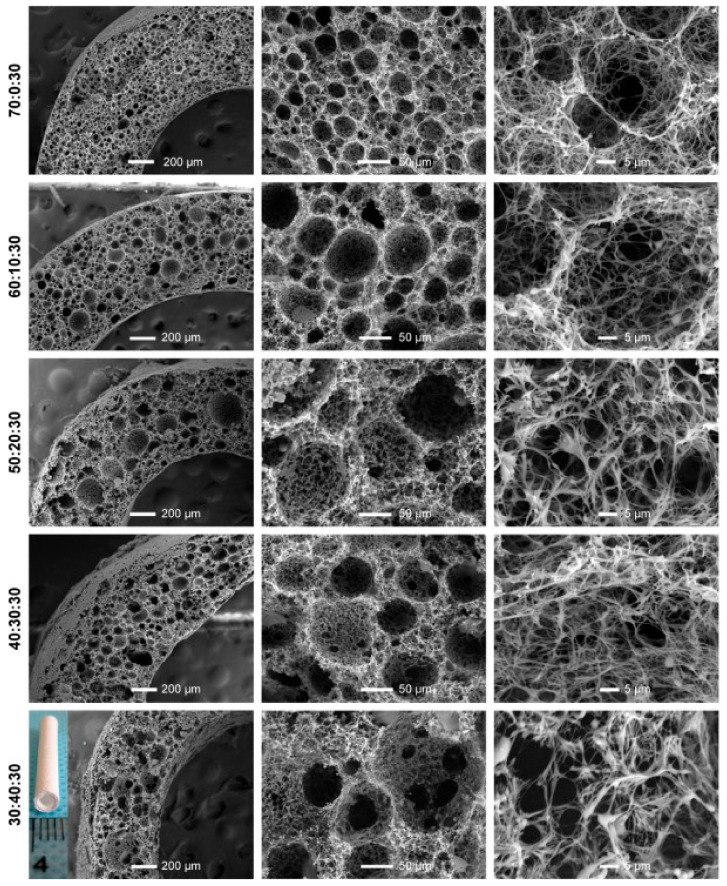
The SEM images and pore diameter distributions of PLLA/PLGA/PCL composite scaffolds with various weight ratios. Reprinted with permission from Dove Press [161].

Nowadays, the most common clinical solution for a heart attack caused by atherosclerosis is vascular bypass grafting. However, this kind of surgery only bypasses a blocked and damaged vessel without repairing the damage caused to heart tissue [172]. Therefore, tissue engineering has focused on creating vascular networks by using scaffolds that could resemble the structure and function of natural vascular tissue. Arterial vessels are composed of a three-layered structure: the outer layer (adventitia) is composed of connective tissue; the middle layer, called media, is composed of smooth muscle cells and extracellular matrix (collagen, elastin, and proteoglycans); the inner layer, called intima, supports a monolayer of endothelial cells [56]. Therefore, producing a functional substitute for such a complex structure is still a challenge. Conventional studies on vessel TE have investigated single-layer scaffolds that mimic only one of the three layers of blood vessels. Reconstruction of the tunica media for vascular tissue engineering was investigated using PLLA/PLGA/PCL hybrid scaffolds [161] (Figure 13).

These scaffolds were seeded with human vascular SMCs (HVSMCs) and evaluated for cell growth and infiltration capacity. The HVSMCs gradually spread on the scaffold surface and proliferated with increasing culture time. After seven days of culture, infiltration of HVSMCs into the interior of the PLLA/PLGA/PCL scaffold was detected, and the expression of the marker protein α-smooth muscle actin (α-SMA) was strongly induced.

Recently, multi-layer scaffolds have been produced for blood vessel TE. For instance, PCL, collagen, and PLLA nanofibers were used to mimic the tunica intima, tunica media, and tunica adventitia, respectively. For this purpose, these nanofibers were fabricated by sequential electrospinning to form a three-layered tubular scaffold [85]. Both endothelial cells and smooth muscle cells were cultured for bioactivity evaluation, showing that the collagen in the middle layer significantly improved attachment and proliferation of SMCs and that endothelial cell proliferation considerably increased with culture, indicating the non-cytotoxicity of the constructs.

Future research directions should focus on the implantation into bigger mammalian models, such as canine or porcine models, and the scaffold seeding by using cells from the patient’s own body.

### 6.4. Skin Tissue

Skin ECM is constituted by fibrous proteins, polysaccharides, two dense tissue layers (i.e., dermis and epidermis), and cells (mainly epithelial cells, keratinocytes, and fibroblasts). When skin incurs severe damages, such as in cases of exposure to high heat or pressure, it cannot self-repair [6,173]. Since the supply of transplantable functional skin is inadequate, significant efforts have been made in the TE field for the development of engineered constructs that facilitate skin repair. In this context, PLLA-based scaffolds have been fabricated and used with cells to form human skin equivalents. Lu et al. [65] took advantage of the mechanical strength of PLLA woven mesh to produce hybrid scaffolds with funnel-like collagen or gelatin sponge. The in vitro dermal fibroblast culture showed that hybrid scaffolds induced high cell seeding efficiency and improved fibroblast adhesion and proliferation compared to control collagen sponge. On the other hand, the in vivo wound healing assessment indicated that the healing occurred faster and more efficiently in the hybrid scaffolds than in the control collagen ones. Mostly, PLLA, collagen, and gelatin have been frequently combined to fabricate nanofibrous scaffolds, showing physical properties and biological characteristics that match those found in skin substitutes [58,138].

PLLA has also been used to produce a multilayered scaffold that closely resembles the natural skin structure. Lou et al. [110] developed a novel bilayer scaffold comprising a superficial chitosan/PCL nanofiber mat (CP-nano mat) and an underlying PLLA microporous disk (PLLA-microdisk). In this work, keratinocytes and fibroblasts were co-cultured as epidermal equivalent and dermal equivalent, respectively. The results showed that cell proliferation was higher in bilayer scaffolds than in single CP-nano mats and PLLA-microdisks. In addition, gene and protein expression evaluations indicated active wound healing, again confirming that the bilayer scaffold could provide a suitable microenvironment for stimulating skin regeneration. Further research should be conducted to generate three-dimensional tissues that meet the clinical application requirements.

## 7. Conclusions and Future Challenges

A biomaterial for tissue engineering applications should possess adequate mechanical properties and suitability to be easily processed. PLLA is a synthetic polymer possessing these prerequisites, and PLLA-based scaffolds have been proven to promote tissue ingrowth and the development of functional substitutes, both in vitro and in vivo. PLLA is an eco-polymer because it is synthesized using green solvents and catalyzers. Nevertheless, the use of PLLA in TE addresses some challenges related to the release of acidic byproducts and their accumulation, which can generate inflammatory conditions, negatively affecting tissue regeneration. Similarly, many attempts have been made to increase the surface hydrophilicity of this polymer and then improve the adhesion of cells and protein absorption. In this review, we describe various strategies adopted to overcome these issues, such as surface modifications and the use of composite constructs made by PLLA and bioactive materials. PLLA is a promising biomaterial for both soft and hard tissue repairs thanks to its relatively slow degradation, as well as its tunable mechanical properties via blending with other polymers or combining with inorganic fillers. Different fabrication technologies have been reported for PLLA-based scaffold manufacturing, such as electrospinning, phase separation, salt leaching, and additive manufacturing, producing structures in various geometries and morphologies both on the macroscale and microscale. From this contribution, it can be stated that PLLA is a biodegradable synthetic polymer possessing a remarkable role in in vitro and in vivo models of tissue engineering, and the review has investigated the recent advances of PLLA-based scaffolds in the biomedical field.

Although PLLA has exhibited great potential for artificial tissues, its clinical application is still limited. In the future, the clinical applicability of the PLLA-based engineered constructs should be assessed by conducting more in vivo studies and clinical trials. A scaffold with functional properties in vitro may cause undesired effects in vivo since the latter includes all the biological and physical stimuli that dynamically change during the phases of tissue repair [174]. Hence, various mechanical and chemical factors, as well as changes in scaffold properties during cell proliferation, should be considered before building a tissue support to better mimic the native tissue [175]. Moreover, the mechanical properties and degradation behavior of the PLLA material are influenced by its crystallinity degree, which is associated with the scaffold fabrication techniques [10]. Researchers must consider all the technical parameters during scaffold fabrication to improve the properties of scaffolds. Finally, more studies on the loading of drugs or antibiotics within PLLA-based scaffolds should be performed for treating associated infections and assessing the pharmacodynamic and pharmacokinetic behavior of the implant.

## Figures and Tables

**Table 1:** Fabrication techniques used for producing PLLA-based scaffolds.

Technique	Processing Parameters	Properties	Advantages	Disadvantages	Reference
Phase Separation (TIPS) and (DIPS)	PLLA concentration Non-solvent concentration Solution thermal history	E = 0.1–12.5 MPa MP = 87–93% Degradation = 3–78 weeks	Low production cost Controlled pore size and porosity	Use of organic solvents	[25,51,63,83,88,89,90,91,92,93,94,95,96,97,98,99]
Porogen leaching	Porogen concentration Porogen size Porogen shape	E = 0.4–81 MPa MP = 69–71% Degradation = 12 weeks	Controlled pore size and porosity Low production costs	Use of organic solvents Low pore interconnection	[12,24,51,95,100,101,102]
Electrospinning	PLLA concentration Applied voltage Solution flow rate Needle-collector distance	E = 1.5–20 MPa TS = 1.5–7 MPa MP = 80–93% Degradation = 1–12 weeks	High porosity Nano-sized fibers High specific surface	Use of organic solvents Low cell intrusion	[22,84,85,103,104,105,106,107,108,109,110,111,112]
Freeze drying	PLLA concentration Solution thermal hystory	E = 40–55 MPa TS = 0.3–5 MPa MP = 73–93% Degradation = 4–12 weeks	Controlled pore size and porosity Low production costs Easy to operate No toxic solvents	Slow production	[65,66,108,113,114,115]
Additive manufacturing	PLLA flow rate Needle dimension Applied temperature	E = 17.2–40.8 MPa MP = >90% Degradation = 24 weeks	Complex structures No toxic solvents High reproducibility	Micro-sized fibers Large pore size	[11,23,107,115,116,117,118,119,120]

**Table 2:** Summary of PLLA-based scaffold materials and properties fabricated for specific TE applications.

Application	Scaffold Material	Fabrication Technique	Properties	Reference
Bone tissue	PLLA/CNT/HA	TIPS	E = 12.5 MPa MP = 87%	[63]
	PLLA	TIPS+PL	MP = 97%	[97]
	PLGA/PLLA/PDLA	Electrospinning	E = 1.55 MPa TS = 2.04 Mpa	[104]
	PLLA/PCL	TIPS	Degradation = 3 weeks	[93]
	PLLA/HA	Electrospinning	MP = <90%	[105,106]
	PLLA/HA	AM	E = 17.2–40.8 MPa MP = >90% Degradation = 24 weeks	[121]
	PLLA/HA	TIPS	E = 3–5 MPa MP = >90%	[98]
	PLLA/HA	PL	E = 0.4 MPa	[103]
	PLLA/HA	TIPS+PL	E = 37–56.2 KPa MP = 88–98	[96]
	PLLA/BG	TIPS	E = 6–8 MPa MP = 88.5% Degradation = 28 days	[90]
	PLLA/BG	PL	E = 55–81 MPa TS = 2.7–4.2 MPa MP = 69–71% Degradation = 90 days	[24]
	PLLA/PCL/HA	PL + freeze drying	E = 1–2 MPa TS = 0.2 MPa MP = 90% Degradation = 78 weeks	[101]
Cartilage tissue	PLLA/PCL	TIPS	E = 90–110 kPa MP = 93–95%	[94]
	PLLA	TIPS	MP = 93%	[84]
	PLLA/chitosan	Freeze-drying	TS = 0.3–0.5 MPa MP = 73–93% Degradation = 28 days	[114]
	PLLA/SF	Electrospinning	TS = 1.5 MPa Degradation = 12 weeks	[110]
Blood vessels	PLLA/gelatin	Electrospinning	E = 6–20 MPa TS = 5–7 MPa MP = 73–75% Degradation = 7 days	[85]
	PLLA/PCL/collagen	Electrospinning	MP = 20–80%	[86]
	PLLA/chitosan/collagen	Electrospinning	TS = 2.13 MPa	[112]
Skin tissue	PLLA/collagen	Freeze-drying	E = 43–55 MPa TS = 5 MPa	[65]
	PLLA/gelatin	Electrospinning	TS = 20 MPaMP = 72%	[22]
	PLLA/gelatin	Freeze-drying	E = 41–43 MPa TS = 5.7 MPa Degradation = 12 weeks	[65]

Abbreviations: TIPS = thermally induced phase separation; PL = porogen leaching; AM = additive manufacturing; E = Young’s modulus; TS = tensile strength; MP = mean porosity.
